# Effect of Layer Thickness in Layered Aluminum Matrix Syntactic Foam

**DOI:** 10.3390/ma12244172

**Published:** 2019-12-12

**Authors:** Chenhao Qian, Chen Liang, Ziyang He, Weixi Ji

**Affiliations:** 1 School of Mechanical Engineering, Jiangnan University, Wuxi 214122, China; 2 Jiangsu Key Laboratory of Advanced Food Manufacturing Equipment and Technology, Jiangnan University, Wuxi 214122, China; 3 School of Engineering, University of Liverpool, Liverpool L69 3GH, UK; Chen.Liang@liverpool.ac.uk; 4 Department of Computer Science, Columbia University, New York, NY 10027, USA; zh2330@columbia.edu

**Keywords:** peak stress, impact ductility, impact failures, layered syntactic foam

## Abstract

This work experimentally investigates the effect of layered structure on the static and impact response of a new layered syntactic foam developed for impact energy absorption. The layered syntactic foam had the same density of 1.6 g/cm^3^ and the same components of 50% large spheres (L) and 50% small spheres (S) with different structures from two layers to five layers. The impact response and energy absorption were investigated by drop-weight impact tests. Under static loading, more layers led to higher yield stress and lower energy absorption. There were three types of progressive failures of layered syntactic form under impact loading. The failure propagation was examined and found to be dependent on the layer number and impact energy. Interestingly, layered syntactic foam absorbed more energy than both of its components in terms of ductility. The ductility of layered syntactic foam decreased with the increase in layer number. The peak stress of layered syntactic foam increased with the increase in layer number. Two-layered syntactic foam LS had the highest ductility under 60 J/g impact, as well as an energy absorption of 35 J/g, compared to other layered syntactic foams. Specifically, its component L had a ductility under 70 J/g and an energy absorption of 25 J/g, while component S had a ductility under 10 J/g and an energy absorption of 10 J/g.

## 1. Introduction

Aluminum matrix syntactic foams (AMSFs) are novel lightweight composites, which consist of an aluminum matrix embedded with ceramic microballoons such as alumina cenospheres [1,2] and fly ash [3,4]. The microballoons are used to introduce porosity in order to form hollow particles inside the AMSFs. These lightweight materials can offer superior specific stiffness, strength, and damage tolerance due to their mechanical energy absorption capabilities. These advantages supply AMSFs with a wide range of applications such as cores in sandwich structures, crash protection, and damping panels [5]. With regard to porous metallic foams, the energy absorption property is generally influenced by the porous structure. It is possible to design specific syntactic foams to meet application demands with different hollow spheres, which can be varied with different densities and sizes using flotation methods and sieves [6].

The mechanical properties of AMSFs with a homogeneous structure were widely studied. Zhao and Tao [7,8] fabricated AMSFs using the infiltration method and studied the effect of Al volume percentage on the compression and energy absorption properties. The confined compression response was also analyzed, as well as the failure mechanisms in AMSFs [9]. Altenaiji [10,11] investigated the impact response using both experiments and simulations. Tao tested the mechanical performance of AMSFs with bimodal ceramic microspheres [12] and toughened Al particles [13].

In addition to a homogeneous structure, functionally graded syntactic foams (FGSFs) are also advanced materials. At least one of their properties can be gradually changed while controlling the position. Therefore, FGSFs show an increasing demand in different industries [14,15,16]. In order to easily control and adjust the properties of syntactic foams to the desired level, it is vital to keep the content of each layer independent during manufacturing. One traditional solution is to prepare each layer independently and then combine them with an adhesive-like epoxy. However, the samples manufactured by this method have poor structural integrity between layers, compared to graded samples which are overall manufactured. Currently, different layers of FGSFs can be produced during one process with infiltration casting, which provides better structural integrity and mechanical performance [17]. The effect of impact behavior on the energy absorption of these materials was studied [18,19,20,21,22]. However, as an important mechanical property in the impact test, the ductility under impact loading was rarely studied, as well as the energy absorption within the ductility of syntactic foam.

This study aims to propose a new layered syntactic foam with high ductility and energy absorption capacity. The effect of the layered structure on the mechanical properties of syntactic foams was studied. Four types of layered syntactic foams (LS, LSL, SLS, and LSLSL, where L = large sphere and S = small sphere) were subjected to quasi-static and impact loading. The layered syntactic foams were made of the same components of 50% L and 50% S but different layer numbers. The mechanical properties and static/impact energy absorption of syntactic foams were experimentally examined.

## 2. Materials and Methods

**Figure 1:**
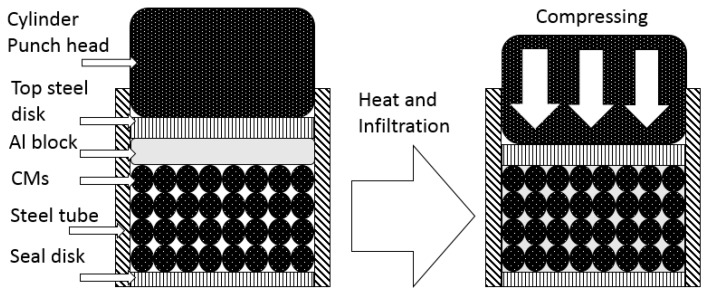
Illustration on infiltration casting.

**Figure 2:**
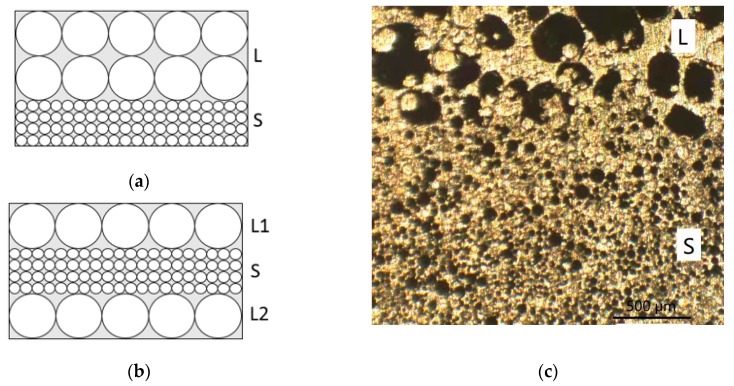
Illustrations and images on layered aluminum matrix syntactic foams (AMSFs): (**a**) LS (L = large sphere; S = small sphere); (**b**) LSL; (**c**) OM (optical microscopy) images.

The AMSF samples were produced by infiltration casting using 6082 Al alloy and a hollow ceramic microsphere (CM) powder supplied by Envirospheres Pty Ltd. (Sydney, Australia). As shown in Figure 1, the CM powder had a composition of ~60% SiO_2_, ~40% Al_2_O_3,_ and 0.4%–0.5% Fe_2_O_3_ by weight, and it was separated into two powders with particle size ranges of 75–150 μm and 250–500 μm, designated as large (L) and small (S), respectively. The two CM powders had a similar density of 0.66 g/cm^3^. Before infiltration, a steel tube, sealed by a circular steel disc at the bottom, was filled either with one layer of the same CM powder or with two or three layers of different CM powders. An Al alloy block was then placed on top of the CM powder(s), and another circular steel disc was placed above the Al block. The assembly was heated to 755 °C for 30 min in an electric furnace before being moved to a hydraulic machine, where the molten Al alloy was compressed into the voids between the CM particles. After solidification, the resultant AMSF sample was removed from the steel tube and ground into cuboid specimens with dimensions of 15 mm × 15 mm × 15 mm for quasi-static compression tests and 10 mm × 10 mm × 15 mm for impact tests. The homogeneous AMSF specimens were designated as L and S, and the layered AMSFs were designed with 2–5 layers, as LS, LSL, SLS, and LSLSL. All kinds of layered AMSFs had approximately 50% L and 50% S. All AMSF specimens had a density of approximately 1.6 g/cm^3^, containing 55% CM particles. The layered structure is shown in Figure 2.

Quasi-static tests were conducted on an Instron testing machine (4045, Norwood, U.S.A.) with a strain rate of 0.001/s up to a stain of approximately 0.7. The specimens were lubricated with oil to reduce friction between the specimen and the platens.

**Figure 3:**
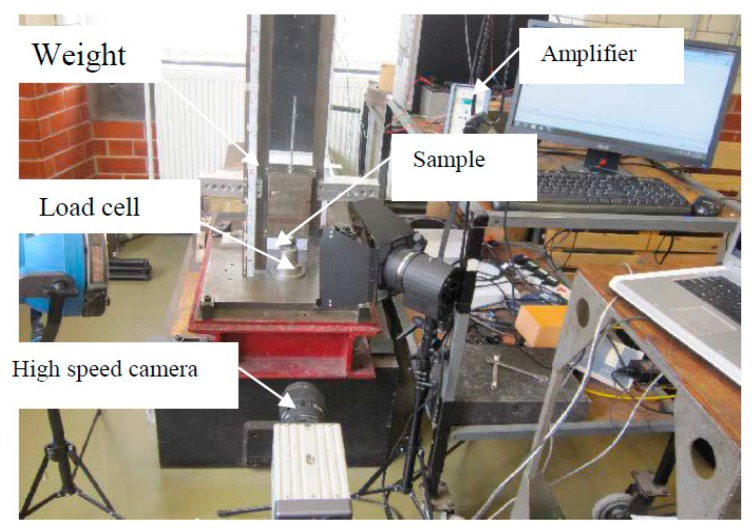
Drop-weight test apparatus.

Impact tests were conducted using an instrumented drop-weight tower, as shown in Figure 3. The specimen was supported by a solid steel base. A hammer with a mass of 15 kg, attached to a carriage guided by two vertical steel bars, was raised to a height varied between 0.2 and 1.2 m to give an incident energy between 10 and 70 J/g with respect to different AMSFs. Table 1 shows the incident energy for each sample. A Kistler 9061A piezo-electric load cell, with a maximum capacity of 200 kN, was used to measure the force–time history. The impact force signal was recorded using the Data Flow Plus software (v 1.0). The hammer velocity and displacement were measured using a MotionPro-X4 high-speed camera at a frame rate of 5000 fps and analyzed using the ProAnalyst software (v 1.0).

## 3. Results

**Figure 4:**
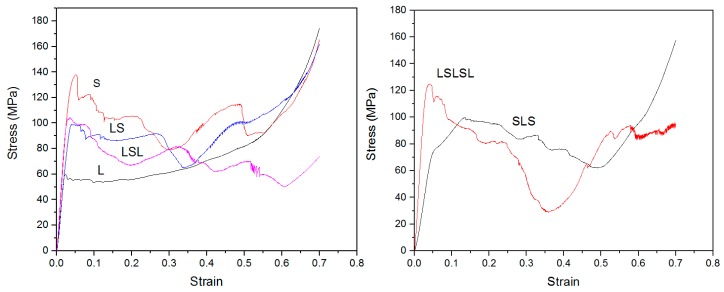
Compressive stress–strain curve of AMSF samples.

Figure 4 shows compressive stress–strain curves of AMSF samples. As shown, L had a low strength 60 MPa and S had a high strength 140 MPa. Layered samples LS, LSL, and SLS had a strength of 100 MPa, which was the average of L and S. LSLSL had a strength of 120 MPa, higher than the average of L and S. With the increase in layer number, the layer relative thickness reduced in size, which led to a smaller barreling effect and higher strength [17]. This could explain the higher strength in LSLSL than the other layered samples. Apart from strength, L had a high ductility with no strength drop during compression, while S had a low ductility with cracking initiated during compression.

Table 2 shows the compressive property of AMSFs; all samples had a porosity of 55%. LS, LSL, and SLS had the average yield stress of L and S, i.e., 100 MPa. LSLSL had a higher yield stress of 125MPa. LS, LSL, and LSLSL had the average Young’s modulus of L and S, i.e., 3 GPa. SLS had a lower Young’s modulus of 1.8 GPa. Generally, the energy absorption in layered AMSFs was the average of L and S. However, LS had the highest energy absorption of 25.5 J/g, while LSLSL had the lowest energy absorption of 21.9 J/g. Such a difference was caused by ductility, as brittle AMSFs tended to crack apart with low energy absorption. A similar behavior was also seen in the impact tests.

**Figure 5:**
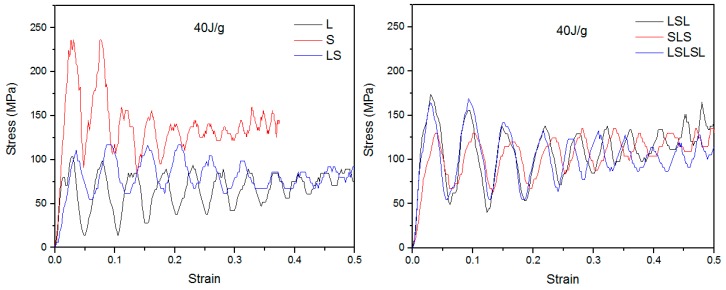
Impact stress–strain histories of AMSFs.

Figure 5 shows the typical stress–strain curves of all AMSFs under 40 J/g impact loading. Stress under impact had a bigger fluctuation than that under compressive loading. Generally, impact loading led to higher stress. In uniform AMSFs, L had a peak stress of 100 MPa, while S had a peak stress of 240 MPa. In layered AMSFs, LS had a lower peak stress of 110 MPa, SLS had a medium peak stress of 126 MPa, and LSL and LSLSL had higher peak stresses of 170 MPa and 165 MPa, respectively. It can be noted that the peak stresses of LS and SLS were significantly lower than the average of L and S (170 MPa). The lower peak stress led to a higher ductility, which is discussed later. The Charpy impact test curves (Figure S1) can also confirm this result.

**Figure 6:**
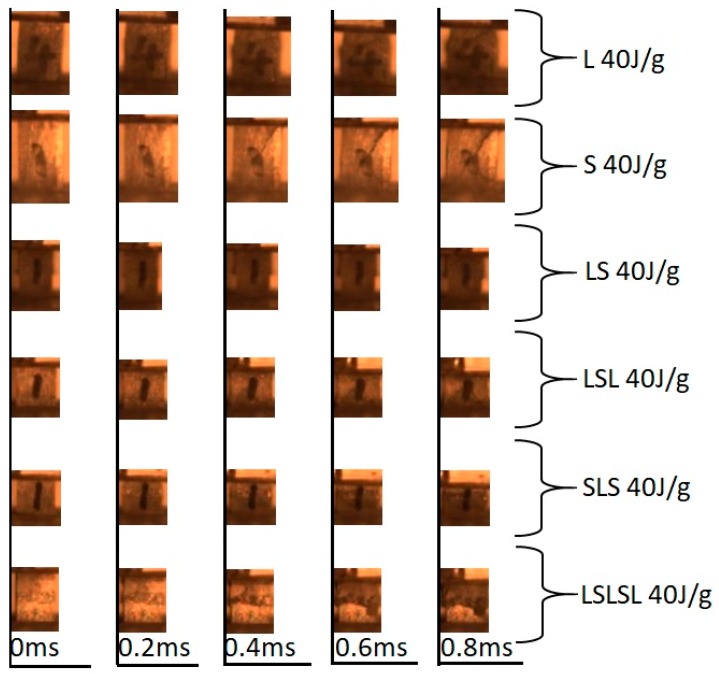
Impact failure sequence.

Figure 6 shows the first five images captured by the high-speed camera for all AMSFs under 40 J/g impact, where the time gap between each frame was 0.2 ms. In L, LS, and SLS, no crack was seen, and the sample was crushed layer by layer. In S, LSL, and LSLSL, a crack was seen in S at 0.4 ms. If we refer to no crack as ductile (D), a crack in the whole sample as brittle (B), and crack in part of the sample as ductile/brittle (DB), then the effect of layer thickness was as shown in Table 3.

Since all layered AMSFs had the same components of 50% L and 50% S, we studied the effect of relative layer thickness on impact ductility, peak stress, and maximum energy absorption within ductility (E_max_). As shown in Table 3, generally, with the increase in impact energy, all AMSFs turned from ductile to brittle. L had excellent ductility up to 70 J/g, while S had poor ductility of 10 J/g. Note than the energy absorption capacity in the compressive test was 20.3 J/g for L and 32.1 J/g for S. This suggests that, although S had higher energy absorption capacity, S could not fulfil such a capacity as it would crack under 20 J/g impact. On the other hand, although L had excellent ductility, the energy absorption capacity of L was low. Thus, the layered AMSFs combined the strength of L and S for an ASMF with higher energy absorption capacity and ductility to fulfil such a capacity.

**Figure 7:**
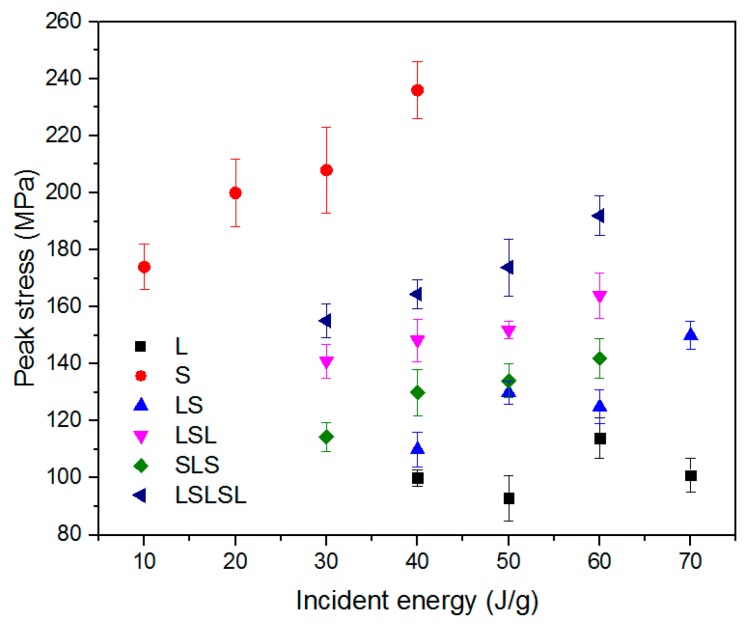
Peak stress under impact in layered AMSFs.

Under impact loading, ductility decreased with the decrease in L thickness. Based on Johnson’s theory [23], impact waves in a thinner layer have more reflections than in a thicker layer, and stress increases with every reflection, which leads to higher stress in the thinner layer. This could also explain the higher strength in LSLSL apart from the barreling effect. Accordingly, peak stress in AMSFs increased with the decrease in L thickness. Peak stress also increased with the increase in impact energy, leading to brittleness. It can be noted that with the 50%–50% component, the peak stress of layered AMSFs was closer to L as opposed to the intermediate value between L and S based on the rule of mixture (ROM), as shown in Figure 7. This suggests that the layered structure led to lower peak stress and higher ductility.

E_max_ in Table 3 shows the energy absorption for each AMSF’s maximum ductility under impact loading. L maintained ductility up to 70 J/g with 25 J/g E_max_, and S was brittle after 20 J/g with 10 J/g E_max_. Layered AMSFs had higher E_max_ than either L or S, and E_max_ decreased with L thickness. LS had the highest ductility of 60 J/g, as well as the highest energy absorption of 35 J/g. This suggests that layered AMSFs combined the high ductility in L and high energy absorption capacity in S, providing both ductility and energy absorption. The two-layered structure was the best choice with the highest ductility under 60 J/g and E_max_ of 35 J/g in all layered AMSFs.

## 4. Conclusions

Layered aluminum matrix syntactic foams (AMSFs) were produced with two sizes of hollow ceramic microsphere—large (L) and small (S). These particles were vertically separated to form a layered structure with 2–5 layers. For comparison, uniform AMSF samples with either large spheres (L) or small spheres (S) were manufactured. The density of all AMSFs was approximately 1.6 g/cm^3^, containing 55% CM particles.

The AMSFs were tested under both compressive loading and impact loading. It was found that L had a high ductility but low energy absorption, whereas S had a high energy absorption but low ductility. The layered structure provided a lower peak stress than the average of L and S, leading to higher ductility. The layered structure helped to improve the ductility in S, as well as fulfil the energy absorption capacity in S, which led to both higher ductility and energy absorption. In the layered AMSFs, reducing the layer thickness by separating one layer into thinner layers led to a higher peak stress and lower ductility. This resulted in the superior impact performance of LS, quantified by a ductility and energy absorption higher than the average of its components.

## Figures and Tables

**Table 1:** Incident energy for aluminum matrix syntactic foam (AMSF) specimens. L = large sphere; S = small sphere.

Specimen Label	Incident Energy (J/g)										
L-1	40	S-1	10	LS-1	40	LSL-1	30	SLS-1	40	LSLSL-1	30
L-2	50	S-2	20	LS-2	50	LSL-2	40	SLS-2	50	LSLSL-2	40
L-3	60	S-3	30	LS-3	60	LSL-3	50	SLS-3	60	LSLSL-3	50
L-4	70	S-4	40	LS-4	70	LSL-4	60	SLS-4	70	LSLSL-4	60

**Table 2:** Compressive strength, Young’s modulus, and energy absorption of four studied AMSFs.

Label	Porosity	Yield Stress (MPa)	Young’s Modulus (GPa)	Energy (Up to 50% Strain) (J/g)
L	55% ± 2%	60 ± 3	3 ± 0.2	20.3 ± 1
S	55% ± 2%	140 ± 5	3.2 ± 0.2	32.1 ± 2
LS	55% ± 2%	100 ± 4	2.8 ± 0.2	25.5 ± 1.5
LSL	55% ± 2%	105 ± 5	3 ± 0.2	23.3 ± 1.5
SLS	55% ± 2%	100 ± 5	1.8 ± 0.2	24.6 ± 1.5
LSLSL	55% ± 2%	125 ± 4	3.1 ± 0.2	21.9 ± 1.5

**Table 3:** Effect of layer relative thickness on impact properties of AMSFs. D = ductile; B = brittle.

**L Thickness**	**S Thickness**	**Ductility**						
		**10 J/g**	**20 J/g**	**30 J/g**	**40 J/g**	**50 J/g**	**60 J/g**	**70 J/g**
100%	0% (L)	-	-	-	D	D	D	D
50%	50% (LS)	-	-	-	D	D	D	DB
	25% + 25% (SLS)	-	-	D	D	D	DB	-
25% + 25%	50% (LSL)	-	-	D	D	DB	B	-
17% + 17% + 17%	25% + 25% (LSLSL)	-	-	D	DB	DB	B	-
0%	100% (S)	D	DB	B	B	-	-	-
**L Thickness**	**S Thickness**	**Peak Stress (MPa)**						
		**10 J/g**	**20 J/g**	**30 J/g**	**40 J/g**	**50 J/g**	**60 J/g**	**70 J/g**
100%	0% (L)	-	-	-	100	95	110	101
50%	50% (LS)	-	-	-	110	130	125	150
	25% + 25% (SLS)	-	-	-	114	130	134	142
25% + 25%	50% (LSL)	-	-	141	148	152	164	-
17% + 17% + 17%	25% + 25% (LSLSL)	-	-	152	165	169	186	-
0%	100% (S)	174	200	208	236	-	-	-
**L Thickness**	**S Thickness**	**E_max_ (J/g)**						
		**10 J/g**	**20 J/g**	**30 J/g**	**40 J/g**	**50 J/g**	**60 J/g**	**70 J/g**
100%	0% (L)	-	-	-	24	24	24	25
50%	50% (LS)	-	-	-	34	35	35	-
	25% + 25% (SLS)	-	-	30	34	33	-	-
25% + 25%	50% (LSL)	-	-	30	33	-	-	-
17% + 17% + 17%	25% + 25% (LSLSL)	-	-	30	-	-	-	-
0%	100% (S)	10	-	-	-	-	-	-

## Supplementary Materials

The following are available online at https://www.mdpi.com/1996-1944/12/24/4172/s1, Figure S1: Charpy impact test curves of impact load-time of the samples.
