# Supplementary material for: Effect of Layer Thickness in Layered Aluminum Matrix Syntactic Foam
Source: Materials (Basel). 2019 Dec 12;12(24):4172. doi: 10.3390/ma12244172 (PMC6947444; doi:10.3390/ma12244172)
Supplement: Supplementary file 1 [file materials-12-04172-s001.pdf]

coatings

Article

Supplymentary Materials: The influence of disc surface topography after vapour blasting on friction and wear of sliding pairs under dry sliding conditions

Chenhao Qian [1,2,*,#], Chen Liang [3,#], Ziyang He [4] and Weixi Ji [1,2,*]

[1] School of Mechanical Engineering, Jiangnan University, Wuxi 214122, China
[2] Jiangsu Key Laboratory of Advanced Food Manufacturing Equipment and Technology, Jiangnan University, Wuxi 214122, China
[3] School of Engineering, University of Liverpool, Liverpool L69 3GH, United Kingdom; Chen.Liang@liverpool.ac.uk
[3] Department of Computer Science, Columbia University, New York, 10027, United States; zh2330@columbia.edu
* Correspondence: qianch@jiangnan.edu.cn (C.Q.); jiweixi@jiangnan.edu.cn (W.J.)
# These authors contribute equally.

Received: 17 November 2019; Accepted: 11 December 2019; Published: date

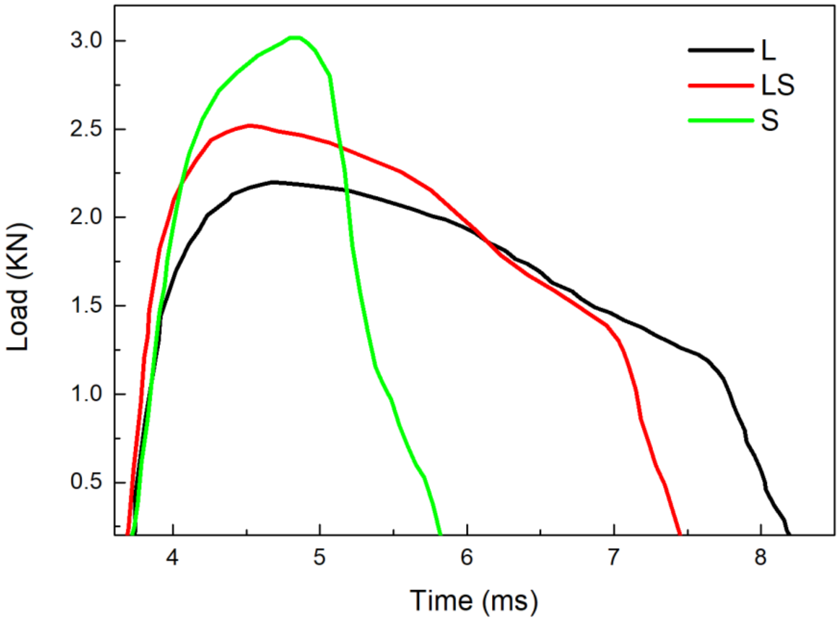

Figure 1. Charpy impact test curves of impact load-time of the samples.

© 2019 by the authors. Submitted for possible open access publication under the terms and conditions of the Creative Commons Attribution (CC BY) license (http://creativecommons.org/licenses/by/4.0/).
